# Supplementary material for: Age-related islet inflammation marks the proliferative decline of pancreatic beta-cells in zebrafish
Source: eLife. 2018 Apr 6;7:e32965. doi: 10.7554/eLife.32965 (PMC5943033; doi:10.7554/eLife.32965)
Supplement: Supplementary file 2. [file elife-32965-supp2.docx]

**Supplementary File 2.**

**List of primer sequences of genes validated using single-cell RT-qPCR and bulk RT-qPCR.**

| **Genes** | **Forward primer** | **Reverse primer** |
| --- | --- | --- |
| insa | TTTAAATGCAAAGTCAGCCACCTCAG | GGCTTCTTCTACAACCCCAAGAGAGA |
| isl1 | AGCAGCAGCAACCCAACGACAA | TGCACCTCCACTTGGTTTGCCT |
| neurod1 | ACGCAGCGCTGTGATATACCGA | TCGCGTTCAACTGGGCGTTCAT |
| tnfα | GCGCTTTTCTGAATCCTACG | TGCCCAGTCTGTCTCCTTCT |
| tnfrsf1b | TCCCATCACAGATGTCAACAC | CTGCTGCTGTGGCTCTGAC |
| ikbaa | CCTGCGTTCCATTCTCACCT | GGCCACTACACTGCTCCTTT |
| ef1a | TCTCTCAATCTTGAAACTTATCAATCA | AACACCCAGGCGTACTTGAA |
| bact2(bact1) | CGAGCAGGAGATGGGAACC | CAACGGAAACGCTCATTGC |
| Rpl13α | TCTGGAGGACTGTAAGAGGTATGC | AGACGCACAATCTTGAGAGCAG |
| socs2 | GCAATCCGAAACCCAAGTC | GCGGAGATGGTGAAGAGGTA |
| gfp | CCTGAAGTTCATCTGCACCA | GGTCTTGTAGTTGCCGTCGT |
| bmi1b | TGAAGATGAGCCTTTGAAAGATTA | GCCTTCTGCCACCACTCATA |
| trib3 | GCGTCTGTTCACTCAGATGG | GTCAGCGAGTCGTCATTTCC |
| cish | AGGGCCCTCACCCTTAACTT | CCGCGGACAGTTTTAACAGA |
| spry4 | CGGATAGACGTCCGCTTTTA | TGGTGAGGAACCCTTGACTC |
| rapgef4 | TGATTGCAAACACAGTCAGAATC | TATCGATCACGCTGATGTGG |
